# CAREGIVING FOR OLDER ADULTS LIVING WITH DEMENTIA AND CONCURRENT HEARING LOSS

**DOI:** 10.1093/geroni/igae098.2527

**Published:** 2024-12-31

**Authors:** Charity Lewis, Wuyang Zhang, Nicholas Reed, Victoria Sanchez, Michelle Arnold, William Haley

**Affiliations:** University of South Florida, Tampa, Florida, United States; Johns Hopkins University, Baltimore, Maryland, United States; Johns Hopkins University, Baltimore, Maryland, United States; University of South Florida, Tampa, Florida, United States; University of South Florida Sarasota-Manatee Campus, Sarasota, Florida, United States; University of South Florida, Tampa, Florida, United States

## Abstract

While hearing loss and dementia are highly concurrent conditions, there is a lack of studies examining its impact on dementia caregiving. Hearing loss has been identified as an unmet need among older adults living with dementia, as it contributes to communication challenges. Using Round 12 (2022) of the linked National Health and Aging Trends Study (NHATS) and National Study on Caregiving (NSOC), we aim to investigate the association of concurrent hearing loss and dementia with caregiving stressors and appraisals. Our study population consisted of 1,445 (Mean age: 61±15 years; Female: 68%) caregivers assisting 942 older adults (Mean age: 82±8 years; Female: 66%; non-Hispanic White: 59%). Hearing loss was measured using pure-tone audiometry and dementia was ascertained using a pre-established algorithm in NHATS. Caregiving stressors, including hours of care in the past month and caregivers’ appraisals of difficulties in physical, emotional, and financial domains, were self-reported. In the analytic sample, the prevalence of hearing loss among care recipients with dementia was high (274/314: 87%). Moreover, 431 (30%) of caregivers provided care to an older adult with concurrent hearing loss and dementia. After adjusting for caregiver-recipient characteristics, results from linear regression models suggest among caregivers assisting older adults with dementia, per 10-dB increment in care recipients’ hearing was associated with a 35% increase in caregiving time (95% CI: 9%-68%). No statistically significant associations were observed regarding caregivers’ perceived difficulties. Our findings suggest that caregivers of older adults with concurrent hearing loss and dementia may be more susceptible to facing greater caregiving intensity.

